# High expression of PTBP1 promote invasion of colorectal cancer by alternative splicing of cortactin

**DOI:** 10.18632/oncotarget.15873

**Published:** 2017-03-03

**Authors:** Zhi-na Wang, Dan Liu, Bin Yin, Wen-yi Ju, Hui-zhong Qiu, Yi Xiao, Yuan-jia Chen, Xiao-zhong Peng, Chong-mei Lu

**Affiliations:** ^1^ Department of Gastroenteology and Hepatology, Peking Union Medical College Hospital, Peking Union Medical College, Chinese Academy of Medical Sciences, Beijing, China; ^2^ Department of Gastroenterology and Hepatology, The Second Affiliated Hospital of Harbin Medical University, Harbin, China; ^3^ National Laboratory of Medical Molecular Biology, Institute of Basic Medical Sciences, Chinese Academy of Medical Sciences and Peking Union Medical College, Beijing, China; ^4^ Department of General Surgery, Peking Union Medical College Hospital, Peking Union Medical College, Chinese Academy of Medical Sciences, Beijing, China

**Keywords:** polypyrimidine tract-binding protein 1, cortactin, colorectal cancer, splicing, invasion

## Abstract

Polypyrimidine tract-binding protein 1 (PTBP1) involving in almost all steps of mRNA regulation including alternative splicing metabolism during tumorigenesis due to its RNA-binding activity. Initially, we found that high expressed PTBP1 and poor prognosis was interrelated in colorectal cancer (CRC) patients with stages II and III CRC, which widely different in prognosis and treatment, by immunohistochemistry. PTBP1 was also upregulated in colon cancer cell lines. In our study, knockdown of PTBP1 by siRNA transfection decreased cell proliferation and invasion *in vitro*. Denovirus shRNA knockdown of PTBP1 inhibited colorectal cancer growth *in vivo*. Furthermore, PTBP1 regulates alternative splicing of many target genes involving in tumorgenesis in colon cancer cells. We confirmed that the splicing of cortactin exon 11 which was only contained in cortactin isoform-a, as a PTBP1 target. Knockdown of PTBP1 decreased the expression of cortactin isoform-a by exclusion of exon 11. Also the mRNA levels of PTBP1 and cortactin isoform-a were cooperatively expressed in colorectal cancer tissues. Knocking down cortactin isoform-a significantly decreased cell migration and invasion in colorectal cancer cells. Overexpression of cortactin isoform-a could rescue PTBP1-knockdown effect of cell motility. In summary the study revealed that PTBP1 facilitates colorectal cancer migration and invasion activities by inclusion of cortactin exon 11.

## INTRODUCTION

Colorectal cancer (CRC) is the third most common malignancy worldwide. Nearly 1.3 million new cases were diagnosed and about 700,000 patients died of CRC per year (Globoscan 2012). According to Cancer Redistry Annual Report in 2013, it became the third most frequently diagnosed and the fifth most leading cause of cancer death in China. CRC are highly heterogeneous, especially in the stage II, with five-year relative overall survival (OS) rates ranging from 87.5% (IIA) to 58.4% (IIC) [1]. Adjuvant chemotherapy are used in stage III CRC patients but in patients with stage II CRC is still a subject of controversy. The understanding of the mechanisms leading to CRC heterogeneity and progression remains elusive.

Alternative mRNA splicing, which allow for combinatorial assembly of different exons from the same primary transcript, is a key mechanism in higher eukaryotes for increasing proteome diversity from a limited gene repertoire. However, in certain pathological conditions such as cancer, aberrantly spliced pre-mRNA can be generated and translated into proteins and which can impair the normal functioning of a cell. Cancer-specific splicing events have been reported at the mRNA level in colon, bladder and prostate tissues [2]. Growing evidences have showed alternative splicing makes great contributions to malignant transformation since they may particularly relevant in the etiology of cancer, provide selective drug targets, or serve as a marker set for cancer diagnosis [3–5].

Polypyrimidine tract-binding protein (PTBP1, also known as hnRNP I) is a member of the heterogeneous nuclear ribonucleprotein family which containing RNA Recognition Motif (RRM) domains and preferentially competes with the splicing factor U2AF65 for binding to polypyrimidine-rich stretches [6–9]. The biological processes that PTBP1 was involved including cell structure and motility, protein targeting and localization, protein metabolism and modification, muscle contraction, cell cycle, immunity and so on [10, 11]. PTBP1 shuttles between nucleus and cytoplasm and initially was described as a key pre-mRNA splicing regulator in alternative splicing [12, 13]. It involved in almost all steps of mRNA metabolism, including transport, mRNA stabilization and initiation of translation in internal ribosome entry site (IRES) as a multifunctional protein is widely accepted [14].

Expression levels of PTBP1 have been found elevated in brain tumors [15, 16], ovarian tumors [17], breast cancers [18] and different malignant cell lines [19]. Furthermore, high expression of PTBP1 has been demonstrated to be associated with aggressive behavior of several types of cancer, especially in glioma and ovarian tumors [16, 17]. A recent study showed that PTBP1 mRNA higher expressed in primary colorectal tumor samples than the normal colonic epithelium and indicated poor prognosis [20]. PTBP1 is the critical determinant of PKM embryonic pyruvate kinase isoform 2, which universally over-expressed in cancer for promoting aerobic glycolysis and may affect drug resistance, in transformed cells [21–25]. PTBP1 also regulates alternative splicing of fibroblast growth factor receptor-1 isoforms [26], alpha-actinin isoforms [27] and multidrug resistance protein 1 (MRP1) isoforms [28], BCL-X alternative 5′ splice site [29], the stability of HIF-1α mRNAs [30] and IRES-mediated translation of p53 isoforms [31]. Thus, PTBP1 palys an important role and has different functions in tumorigenesis by regulating amounts of target genes associated with malignancy. However, there was no evidence of its prognostic significance in patients with stages II and III CRC, and the role of alternative splicing of PTBP1 in CRC tumogenesis was still unknown.

In our research, we demonstrated elevated levels of PTBP1 are associated with poor clinical outcome in stages II/III CRC patients. PTBP1 expression was upregulated both in colon cancer cell lines and primary colorectal cancer. Inhibition of PTBP1 expression in CRC cells decreased tumor cell proliferation, migration and invasion. Knockdown of PTBP1 inhibited CRC cell growth in nude mice. Furthermore, PTBP1 was proved to play an important role in CRC alternative splicing regulation progression, the exon 11 of cortactin gene is one of its target and the alternative splicing influences cell motility in CRC cell lines.

## RESULTS

### The overexpression of PTBP1 in stages II/III CRC patients predicted poor clinical outcome

**Figure 1:**
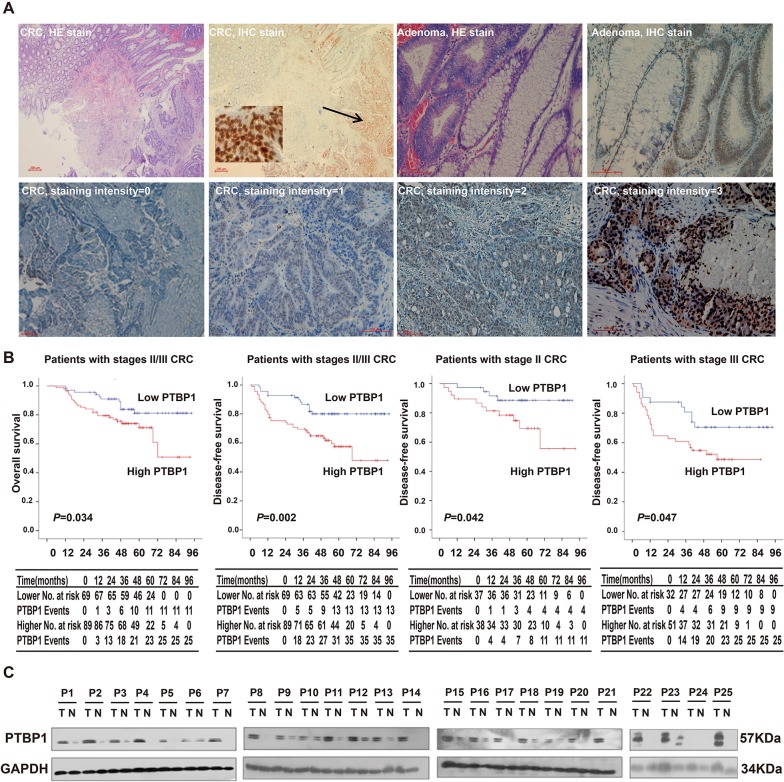
PTBP1 is highly expressed in colorectal cancer and high expression of PTBP1 is associated with poor prognosis **(A)** HE staining and immunohistochemical staining for PTBP1 in human colorectal cancers (CRC) and adenomas. The arrows point to the positive staining of tumor cells. PTBP1 mainly localized in the nucleus of tumor cells in immunohistochemical staining. Staining intensities of CRC were classified as 0 (no staining), 1(weak staining), 2 (distinct staining) and 3 (very strong staining). Scale bar, 200μm (CRC, HE stain and IHC stain) and 100μm (others). **(B)** Overall survival and disease-free survival curves for patients according to the expression levels of immunohistochemical variables in 75 stage II and 83 stage III CRC patients. No. at risk=number at risk; PTBP1 Events=cumulated events (death/relapse). **(C)** Western blot showing PTBP1 protein levels in 25 pairs of CRC tissues and paired normal samples. GAPDH was used as a loading control.

PTBP1 expression levels were determined by using immunohistochemical analysis in 202 CRC, 44 adenomas and 106 normal colonic epitheliums. The localization of the PTBP1 protein was predominantly stained in the nucleus with heterogeneous intensity in each group (Figure 1A). In the study, CRC patients were divided into two groups, low-level group and high-level group based on HSCORE in methods section (HSCORE<2 and HSCORE ≥2, respectively). We found that PTBP1 displayed the more frequent strong immunoreactivity both in colorectal cancer samples (56.3%, 89/158) and adenomas (77.2%, 34/44) which comparing with normal colon epithelium (10%, 11/106) (*P*<0.001, respectively). In order to investigate specifically the prognostic value of high PTBP1 expression level, tissues from patients with stageIcolorectal cancer (very low risk of progression) and patients with preoperatively distant metastases (stage IV) patients were excluded. However, in 158 patients with stages II/III CRC, the expression level of PTBP1 were not related with any of the clinicopathological parameters (Table 1) (the data of 202 CRC patients are shown in Supplementary Table 3) but high PTBP1 expression was more frequently in CRC with nodal involvement (50.7% in Stage II vs 61.4% in Stage III, *P* =0.17).

**Table 1:** Correlation of clinicopathologic features with PTBP1 expression in 158 stages ii/iii patients with colorectal cancers

Clinicopathologic Features	Case	PTBP1 expression		P*
		Low levels(%)	High levels(%)	
Case	158	69	89	
Age,years				
<60	64	31(19.6%)	33(20.9%)	0.32
≥60	94	38(24.1%)	56(35.4%)	
Gender				
Male	92	40(25.3%)	52(32.9%)	0.95
Female	66	29(18.4%)	37(23.4%)	
Tumor site				
Colon	79	30(19.0%)	49(31.0%)	0.15
Rectum	79	39(24.7%)	40(25.3%)	
Tumor size, cm				
<5	82	37(23.4%)	45(28.5%)	0.7
≥5	76	32(20.3%)	44(27.8%)	
Histological type				
Adenocarcinoma	131	56(35.4%)	75(47.5%)	0.61
Others*	27	13(8.2%)	14(8.9%)	
Grade				
G1/G2	120	50(31.6%)	70(44.3%)	0.38
G3	38	19(12.0%)	19(12.0%)	
Tumor status				
T1 and T2	7	5(3.2%)	2(1.3%)	0.261
T3 and T4	151	64(40.5%)	87(55.1%)	
Nodal status*				
N0	75	37(23.4%)	38(24.1%)	0.17
N1/N2	83	32(20.3%)	51(32.3%)	
TNM Stage				
II	75	37(23.4%)	38(24.1%)	0.17
III	83	32(20.3%)	51(32.3%)	

Note: *Others=mucinous or signet-ring cell carcinoma. Tumor grade: G1=well differentiated, G2=moderately differentiated, G3=poorly differentiated. Nodal status: N0=no lymphnode involvement. N1=1-3 nodes involved. N2=more than or equal to four nodes involved. *P* value, Pearson's χ2 test or Continuity Correction test.

In the other hand, high expression of PTBP1 in 158 stages II/III CRC patients were associated with poor OS and disease-free survival (DFS) rate (Figure 1B), similar to the results got from 202 stages I-IV CRC (Supplementary Figure 1). Furthermore, a significant negative correlation relationship showed between DFS and PTBP1 expression levels in 75 stage II and 83 stage III CRC patients, respectively and independently (Figure 1B). In univariate analysis, clinicopathological parameters such as nodal status and high expression levels of PTBP1 were important prognostic factors (Table 2). Taking consideration of the effect of other clinical features, high expression of PTBP1 maintained its significance as an independent prognostic factor for DFS in multivariate Cox proportional hazards model. The high expression levels of PTBP1 indicated a 2.577-fold (95% CI; 1.321-5.025, *P* =0.006) greater risk of relapse or death (Table 2).

**Table 2:** Univariate analysis and Multivariate analyses of overall survival rates and disease-free survival rates in 158 Stages II/III Patients with Colorectal Cancers

Variables	Univariate analysis(log-rank)		Multivariate analysis(Cox model)			
	OS	DFS	OS		DFS	
	*P*	*P*	HR [95% CI]	*P*	HR [95% CI]	*P*
Age, years (≥60/<60)	0.2	0.21	0.906[0.960-1.016]	0.38	0.996[0.973-1.019]	0.748
Gender (Male/Female)	0.55	0.95	1.436[0.709-2.910]	0.315	1.123[0.624-2.021]	0.699
Tumor site (Colon/Rectum)	0.36	0.33	0.613[0.311-1.207]	0.157	0.621[0.348-1.107]	0.106
Tumor size, cm (≥5/<5)	0.46	0.76	1.151[0.568-2.331]	0.698	0.845[0.463-1.543]	0.584
Histological type (Adenocarcinoma/Others)	0.63	0.87	1.089[0.408-2.903]	0.865	1.080[0.439-2.658]	0.868
Grade (G3 /G1-G2)	0.12	0.37	1.887[0.848-4.184]	0.12	1.468[0.725-2.976]	0.286
Tumor status (T3-4/ T1-2)	0.529	0.751	1.938[0.245-7.538]	0.531	1.357[0.306-6.024]	0.688
Nodal status (N1-2/ N0)	0.006*	0.004*	2.710[1.279-5.714]	0.009*	2.273[1.217-4.255]	0.01*
PTBP1 expression (High / Low)	0.034*	0.002*	2.012[0.951-4.255]	0.068	2.577[1.321-5.025]	0.006*

Note: The Cox proportional hazards regression model contained the significantly different factors in univariate analysis (Nodal status and PTBP1 expression) and the common important factors which may affect the survival rate(age, gender, tumor site, tumor size, histological type, grade and tumor status). HR, hazard ratio; CI, confidence interval; * means *P* < 0.05

### PTBP1 is upregulated in CRC and promotes tumor proliferation, migration and invasion

**Figure 2:**
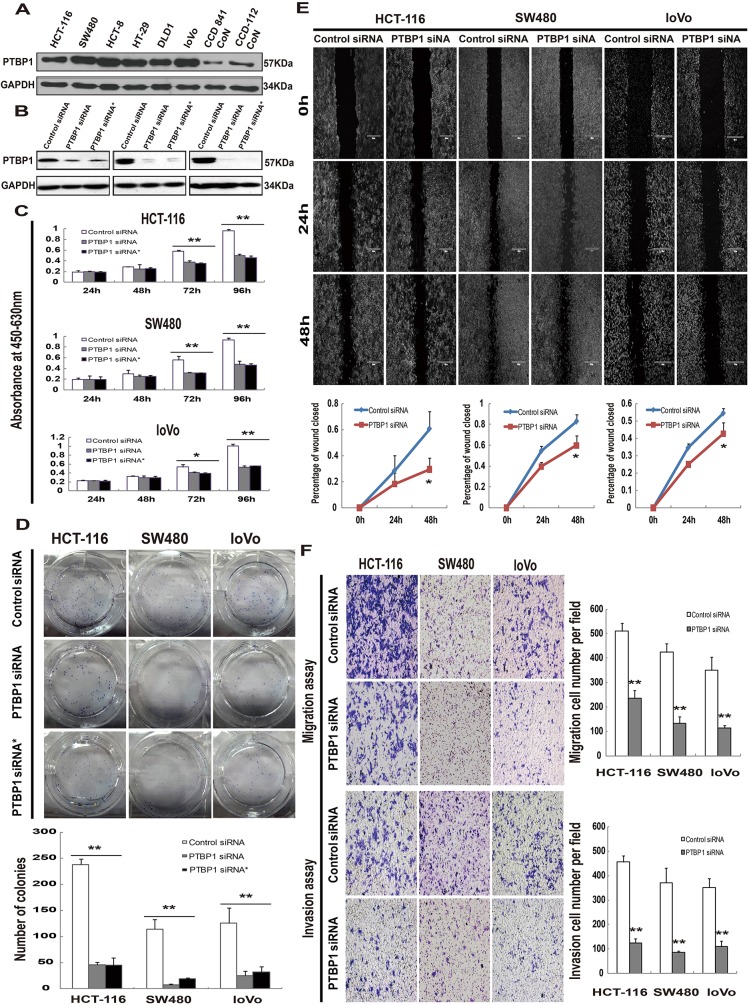
Kockdown of PTBP1 inhibites tumor proliferation and migration/invasion *in vitro* **(A)** Representative Western blot showing PTBP1 protein levels in six human colorectal carcinoma cell lines (HCT-116, SW480, HCT-8, HT-29, DLD1 and loVo)and two human normal colon cell lines (CCD 841 CoN and CCD-112 CoN). GAPDH was used as a loading control. **(B)** Western blot below demonstrating significant siRNA-mediated knockdown of PTBP1 between 48 to 96 hours post-transfection in 3 colorectal carcinoma cell lines (HCT-116, SW480 and loVo). GAPDH was used as a loading control. **(C)** MTT assays were performed to investigate the effect on the growth and survival of on the same 3 colorectal carcinoma cell lines after transfection with the control siRNA or PTBP1 siRNA as above. Data are presented as mean ± SD for four wells and are representative of 3 separate times. *P < 0.05 and **P < 0.01. **(D)** Colony formation assays were utilized to test the colony formation ability in the respective cell lines after transfection with the control siRNA or PTBP1 siRNA. Values represent means ± SD and three repeated experiments were performed. **P < 0.01. **(E)** Wound-healing assay was utilized to test the migration abilities of the 3 cell lines above after transfection. The wound closure was quantified at 24h and 48h post-wound by measuring the remaining unmigrated area using Image-Pro Plus, and percentage of wound closed were calculated (mean ± SD, 3 separate times). *P < 0.05. **(F)** Cell migration and invasion assays were determined after transfection. Photos were taken under inverse microscope (Nikon, Eclipse, TE2000-U), 10×. Data are presented as mean ± SD and are representative of 3 repeated experiments. **P < 0.01.

PTBP1 expression pattern was confirmed by immunohistochemistry, proteins from 25 CRC tissues and paired normal samples were extracted. The results showed that overexpression of PTBP1 in CRC tissues and reduced expression in normal samples (Figure 1C). We also determined protein levels of PTBP1 in 6 different human colon cancer cell lines (HCT-116, SW480, HCT-8, HT-29, DLD1 and loVo), human normal colon epithelial cell lines (CCD 841 CoN) and human normal colon fibroblast cell line (CCD-112 CoN). We found PTBP1 was upregulated in 6 CRC cell lines compared with 2 normal cell lines (Figure 2A), consisting with the results of tissues.

The frequent over-expression of PTBP1 in CRC tissues and colon cell lines but not in normal tissues and cell lines implicated that PTBP1 may have a role in CRC development. To test the speculation, HCT-116, SW480 and loVo cell lines were selected because of over-expression of PTBP1 and 2 siRNAs (PTBP1 siRNA and PTBP1 siRNA*) which specifically target PTBP1 and one control siRNA were synthesized. After transfection for 48 to 72 hours, siRNA-mediated knockdown of PTBP1 was confirmed in protein levels (Figure 2B). Colon cancer cells transient transfected with PTBP1 siRNA caused a significant decrease in cell viability compared with those transfected with the control siRNA. The reduction became more apparent at 72 and 96 hours by MTT assay (Figure 2C). The inhibition of colon cancer cell proliferation was further confirmed by colony formation assay. A significant reduction of colony numbers was observed transfected with PTBP1 siRNA than in the controls (Figure 2D). Moreover, the results of two-color fluorescence-activated cell sorting analysis showed no differences in the numbers of early apoptotic cells but a increase numbers of late apoptotic cells which may affect cell proliferation (Supplementary Figure 2). Thus, the treatments knock down of PTBP1 exhibits growth ability in colon carcinoma cells. To investigate the role of PTBP1 in cell migration and invasion, we conducted Wound-healing assays and Transwell Migration/Invasion assays. The results indicated that PTBP1 knock-down remarkably reduced the migratory and invasive ability of colon cancer cells (Figure 2E and 2F). According to the above results, it is illustrated that PTBP1 knockdown inhibits colon cancer cell growth and migration/invasion.

**Figure 3:**
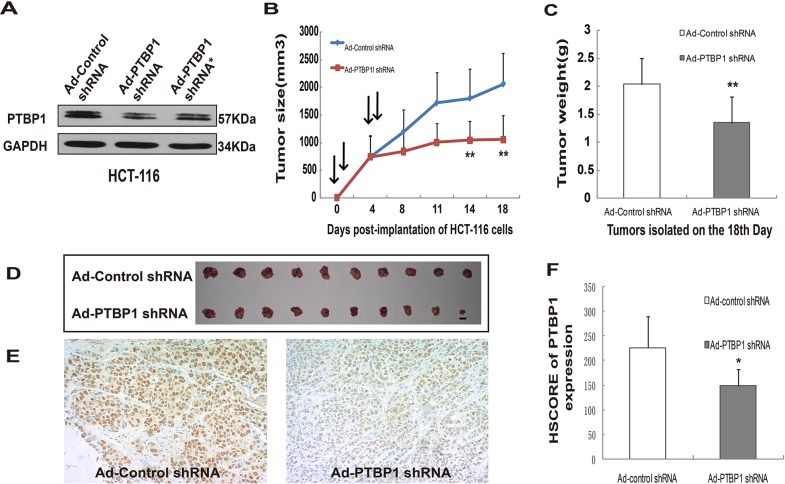
Kockdown of PTBP1 generally inhibited tumor growth *in vivo* **(A)** Representative Western blot showing PTBP1 levels in HCT-116 cells after infection with Ad-shRNA. GAPDH was used as a loading control. **(B)** Growth curves of tumors in nude mices (n = 10 for each group). Arrows represents intratumoral injection of adenovirus. Growth curves represents days after adenovirus injection in x axis and tumor volume (V = (L × W2)/2; L, length;W, width) in y axis. Data form are presented as mean ± SD. **P < 0.01 compared with Ad-Control shRNA group and AD-PTBP1 shRNA group by a Student's t test. **(C)** Tumors were isolated and weights were measured on the 18th day of the two groups above. Data form are presented as mean ± SD. **P < 0.01. **(D)** Subcutaneous tumors isolated from nude mice 18 days after adenovirus injection (n = 10 for each group). Scale bar: 10mm. **(E)** Immunohistochemistry for PTBP1 of the two groups of tumor (×400). **(F)** The HESCORE of PTBP1 expression in the isolated tumors in the two groups. Data form are presented as mean ± SD. *P < 0.05.

In order to show the effect of decreased PTBP1 expression levels in colon carcinoma cells *in vivo*, we synthetized an analogous recombinant adenovirus which encoded a PTBP1 shRNA or control shRNA. AD-PTBP1 shRNA targeted different sites on the PTBP1 mRNA which should have reduced the possibility of an off-target effect. PTBP1 knockdown was confirmed by Western blotting (Figure 3A). Next, we selected one of the AD-PTBP1 shRNA and generated xenografts in immune-compromised nude mice. 10^7^ HCT-116 cells infected with adenovirus expressing PTBP1 shRNA or control shRNA were subcutaneously injected into 20 nude mice in total (each at 10^8^ pPFUs). The first day of adenoviral shRNA injection was recorded as day 0. We repeated intratumoral injections of the same adenoviruses-infected cells on days 1, 4, and 5 and measured the tumor size every 4 days. At the 18th day, these mice were sacrificed and tumor weight was measured. On days 14 and 18, tumors derived from AD-PTBP1 shRNA group were significantly smaller in tumor size and much lighter in tumor weight than the control group when tumors were isolated (Figure 3B, 3C and 3D). PTBP1 expression levels of the two groups were confirmed by immunohistochemistry and HSCORE (Figure 3E and Figure 3F).

### PTBP1 regulates alternative splicing of its many target genes in CRC

**Figure 4:**
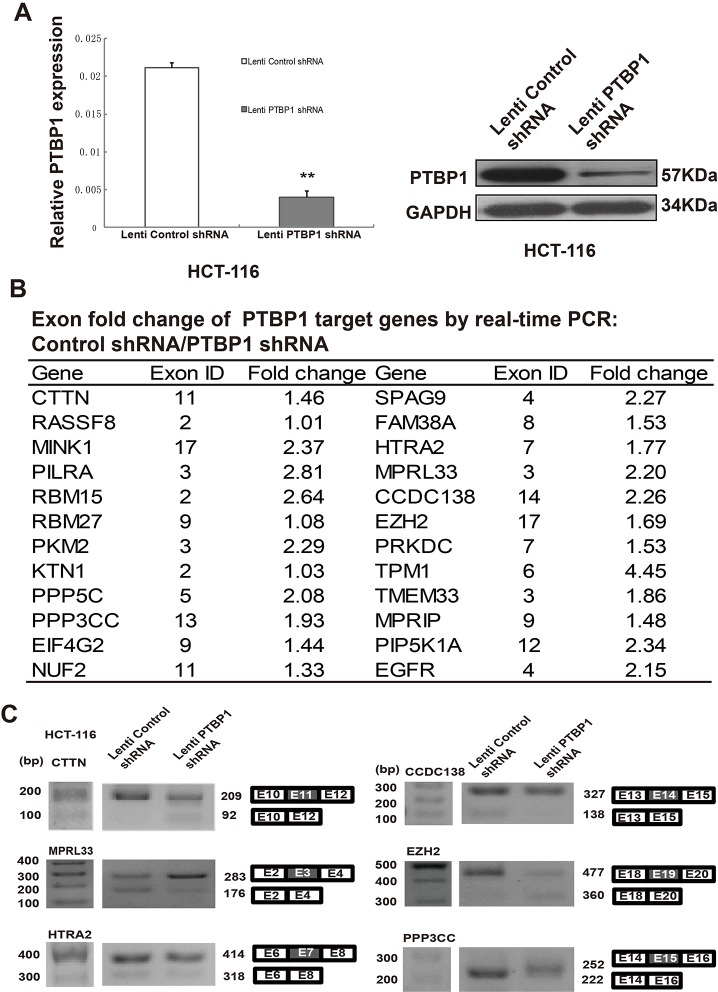
PTBP1 regulates levels of mRNA and alternative splicing in HCT-116 cells **(A)** Real-time PCR and western blot demonstrating significant lentiviral-mediated shRNA-mediated knockdown of PTBP1 for at least 30 days in HCT-116 cell lines. GAPDH was used as a loading control. Data are mean ± SD. **P < 0.01. **(B)** Exon fold change list of target genes between normal condition and PTBP1 knockdown. *Note: Known Gene exon in UCSC genome database (hg18) is according to the following reference: Xue Y, Zhou Y, Wu T, et al. Genome-wide analysis of PTB-RNA interactions reveals a strategy used by the general splicing repressor to modulate exon inclusion or skipping. Mol Cell 2009;36:996-1006. The experiment was repeated three times and data represented as mean ± SD. **(C)** Six target genes of PTBP1 were validated by RT-PCR from 24 selected genes in HCT-116 cells. Diagrams show possible transcripts arising from splicing of exons in CTTN, MPRL33, HTRA2, CCDC138, EZH2 and PPP3CC. White, flanking constitutive exons; gray, PTBP1-activated/repressed exons. Molecular markers are shown to the left of gels.

To define the significant regulation of some PTBP1 target genes, including levels of mRNAs and alternative splicing, we used lentiviral-mediated shRNA targeting PTBP1 and PTBP1 depletion was highly efficient (knockdown 80%) in HCT-116 cells and sustained for at least 30 days (Figure 4A). According to previous study by cross-linking immunoprecipitation coupled with highthroughput sequencing (CLIP-seq) in Hela cells [10], we examined the levels of mRNAs changes of PTBP1 target genes via quantitative real time PCR in HCT-116 cells (Figure 4B and Supplementary Figure 3A). Then we studied how each exon changes of the different target genes by real time PCR in HCT-116 cells (Supplementary Figure 3B). Some of them were also validated by RT-PCR (Figure 4C). The function of these genes included cell structure and motility (TPM1, PIEZO1, MPRIP), protein targeting and localization (CTTN, RASSF8, KTN1), protein metabolism and modification (PILRA, MINK1, PKM2, PPP5C, PPP3CC), apoptosis (EZH2, HTRA2, CASP2), DNA repair (PRKDC) and so on. The final biological effect depended on the combination of different transcripts, transcription levels of mRNAs and the network of amounts of genes.

### PTBP1 mediates alternative splicing of the exon 11 in cortactin pre-RNA

**Figure 5:**
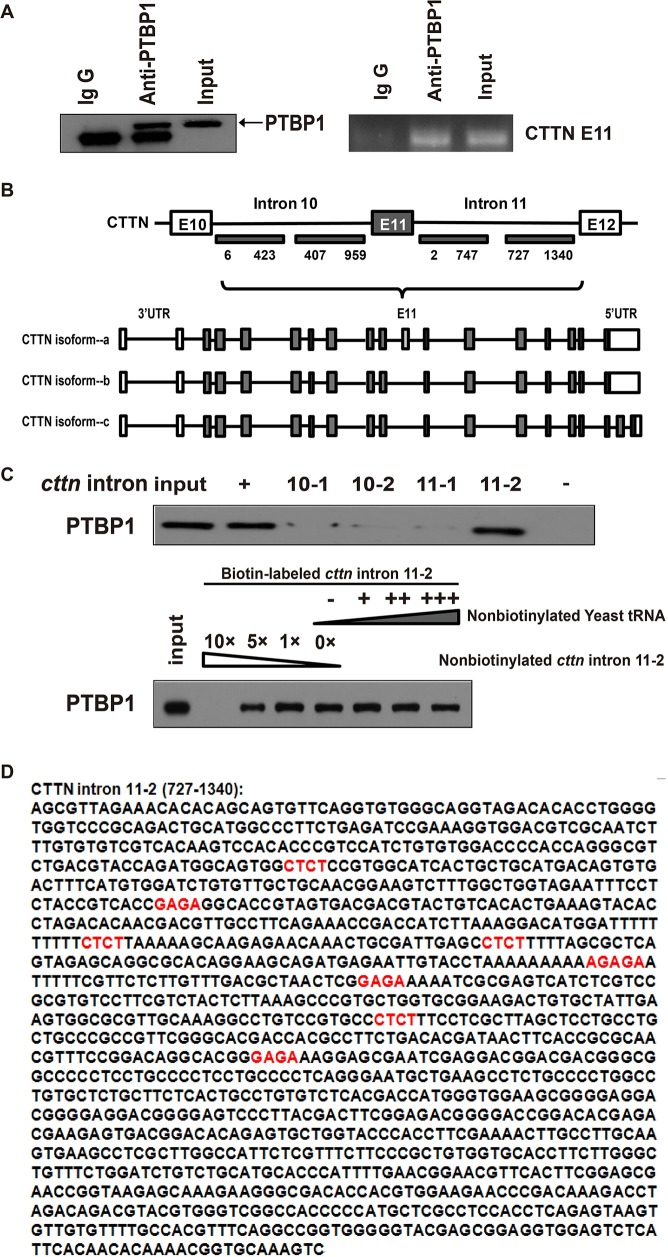
PTBP1 mediates alternative splicing of the exon 11 in cortactin(CTTN) pre-RNA **(A)** CTTN RIP with PTBP1 antibody in HTC-116 cell line. PTBP1 protein precipitation upon RIP. The experiment was repeated three times. **(B)** The mRNA models of 3 cortactin isoforms and the specific fragments of CTTN intron used as templates for the synthesis of biotin-labeled RNAs are indicated with underlines, and the nucleotide positions amplified by PCR are shown. **(C)** A specific association between PTBP1 and CTTN intron 11-2 was confirmed by biotin pull-down analysis. A negative control and EV71-IRES were also used. A competition assay was used to confirmed the specific association between PTBP1 and CTTN intron 11-2 (identified in B). One, Five- or ten-fold excess of unlabeled RNA was added to compete with the biotin-labeled RNA for interaction with PTBP1, and excess of cell lysates was incubated with biotin-labeled RNA. More than 3 repeated experiments were performed. **(D)** Potential PTBP1 binding regions within the CTTN intron 11-2 sequence are indicated by bold red (CUCU).

Given that PTBP1 may regulate cortactin exon 11 which can be seen in RT-PCR (Figure 4C), we performed RNA immunoprecipitation and showed that PTBP1 efficiently coprecipitated cortactin RNA in the colorectal cancer cell line HCT-116, confirming a direct interaction (Figure 5A). Then a biotin pull-down assay was applied for further investigation. The biotin-labeled RNA probes were obtained from the corresponding fragments located in the intron 10 or intron 11 of *cortactin* gene (Figure 5B). The untanslated sequence in mouse genome (nonsense sequence) and the EV71-IRES (human enterovirus 71-internal ribosome entry site) were used as negative and positive controls, respectively. The RNA probe-protein pull down complexes in HCT-116 cell were analyzed by Western blot using an antibody against PTBP1. Intron 11-2 of *cortactin* could specifically bind to PTBP1, but the other 3 different fragments within intron 10 or intron 11 of *cortactin* could not. PTBP1 was not detected in the negtive control complex. In contrast, the EV71-IRES (positive control) showed a strong binding of PTBP1 (Figure 5C). To further confirm the specificity of the binding between PTBP1and the intron 11-2 of *cortactin*, a competition assay was performed. The biotin-labeled RNA was in excess and 1, 5 or 10 fold excess of unlabeled homologous probe was added to compete with the biotin-labeled probe for PTBP1 binding. Strong competition at 10-fold excess of the unlabeled probes exhibited in the results (Figure 5C). All these findings suggested that the major determinant fragment binding PTBP1 resides in the intron 11-2 of *cortactin* (Figure 5D). It has been reported that PTBP1 can bind to CU-rich sequences at polypyrimidine-rich regions of RNA [10, 11, 32, 33]]. We further confirmed several motifs (CUCU) containing potential binding sites within the fragment of intron 11-2 of *cortactin* gene by prediction (Figure 5D).

### The protein expression of PTBP1 is consistent with cortactin isoform-a in colorectal tissues and cells

**Figure 6:**
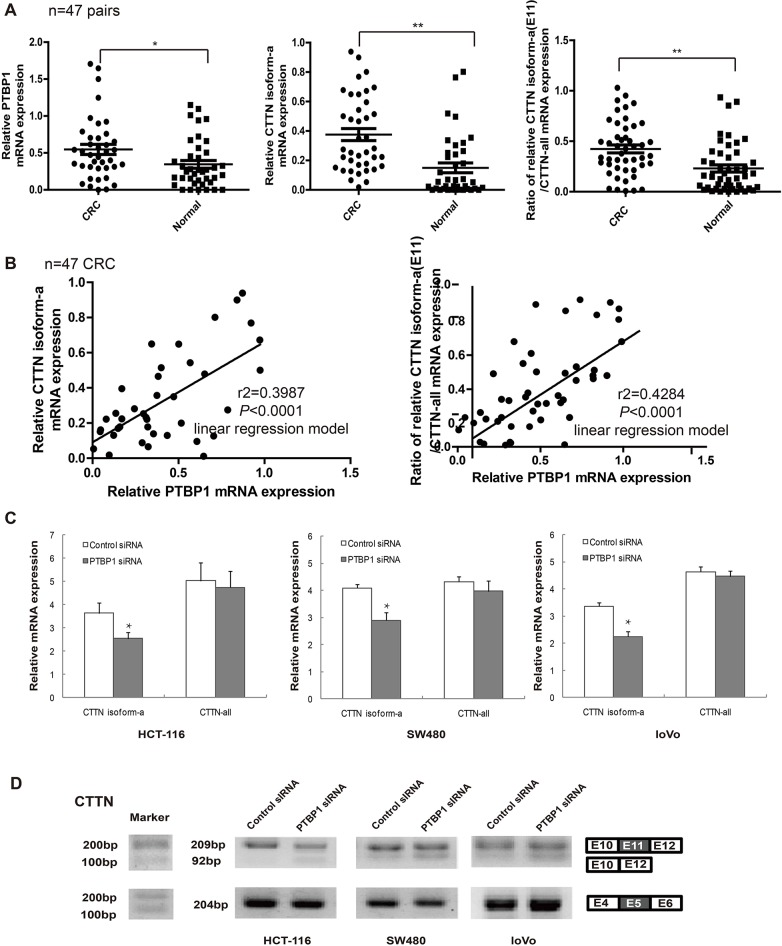
The protein expression of PTBP1 is consistent with that of CTTN isoform-a (E11) in colorectal cancer and cells **(A)** The mRNA levels of CTTN isoform-a (E11) and the ratio of CTTN isoform-a/ CTTN-all mRNA in selected 47 paired colorectal tissues compared to normal colon samples by real-time PCR. RNA input was normalized to GAPDH mRNA. **P < 0.01. **(B)** The mRNA levels of PTBP1 is consistent with that of CTTN isoform-a (E11) and is also positive related with the ratio of CTTN isoform-a/CTTN-all mRNA in 47 CRC tissues by linear regression model. **(C)** The mRNA levels of CTTN isoform-a (E11) and CTTN-all after PTBP1 transiently knock-down in three types of colorectal cancer cells (HCT-116, SW480 and loVo) by real-time PCR. The relative mRNA levels were normalized to GAPDH. Expression data are presented as the mean ± SD of triplicate samples. *P < 0.05. **P < 0.01. **(D)** CTTN exon 11 as PTBP1 target was validated by RT-PCR in the three colorectal cancer cells. CTTN exon 5 as controls (not PTBP1 target). Molecular markers are shown to the left of gels, and the identity of bands is indicated to the right.

Over-expression of *PTBP1* or *cortactin* in mRNA levels and protein levels has been proved the relevance of tumor stage and prognosis in CRC previously [20, 34]. Cortactin (CTTN) isoform-a, which is the only one containing exon 11, is the most among all the cortactin transcripts (Figure 5B). To explore the relation between PTBP1 and cortactin isoform-a, we first detected their mRNA expression levels in colorectal cancer tissues. Real-time PCR showed that mRNA levels of PTBP1 and cortactin isoform-a, and the ratio of cortactin isoform-a to all its transcripts, were both increased in 47 stages II/III CRC comparing with the paired normal colon tissues (Figure 6A). Morever, there was a positive correlation in 47 CRC between PTBP1 and cortactin isoform-a or the ratio of cortactin isoform-a to all its transcripts in a linear regression model (Figure 6B). Then after siRNA-mediated knockdown of PTBP1, cortactin isoform-a was significantly decreased in three colorectal cancer cell lines by real-time PCR and RT-PCR but the total mRNA levels of *cortactin* remained slightly unchanged (Figure 6C and 6D). Above all, these findings confirmed the expression relevance of PTBP1 and cortactin isoform-a in CRC and PTBP1 mediates inclusion of the alternative exon 11 in *cortactin* pre-RNA.

### Over-expression of cortactin isoform-a could rescue PTBP1-knockdown effect of cell motility

As an actin-associated scaffolding protein that regulates cell migration, cortactin has been reported to be overexpressed in CRC [35]. And cortactin isoform-a, which is the wild type and dominant one containing the full functional repeats, has the strongest filamentous actin (F-actin)-binding, cross-linking and cell migration abilities [36]. Cortactin isoform-b and cortactin isoform-c (much less), lacks the 6th repeat (exon 11), show reduced F-actin binding and polymerization ability and significantly reduced cell migration when compared with cortactin isoform-a [36]. To confirm the function of cortactin isoform-a, we first designed a specifically siRNA targeting *cortactin* exon 11, the knockdown effect of exon 11 was confirmed by real-time PCR and RT-PCR after transfection and there was a significant reduction after transfection with cortactin isoform-a siRNA than in the controls by Transwell Migration/Invasion assays (Supplementary Figure 4), which is similar to the results in the PTBP1 knockdown cells (Figure 2E and 2F).

**Figure 7:**
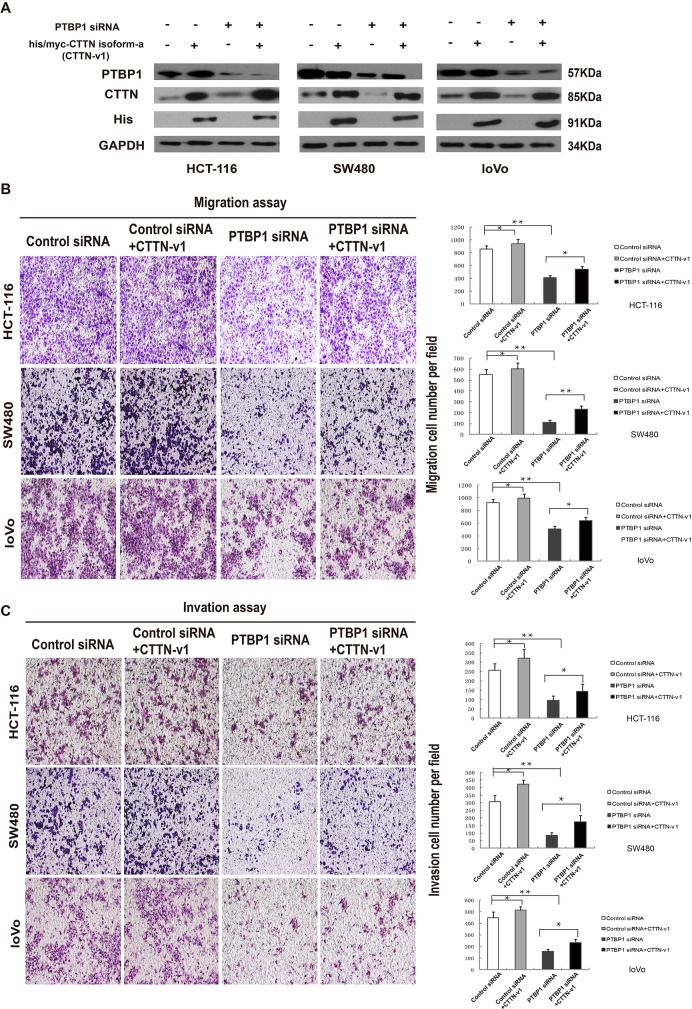
Overexpression of cortactin isoform-a could rescue PTBP1-knockdown effect of cell motility **(A)** Forty-eight hours after co-transfection with PTBP1 siRNA/control siRNA and his/myc-cortactin isoform-a construct (CTTN-v1)/empty vector, colorectal cancer cells were collected, total protein was extracted and western blotting was used to determine the expression of PTBP1 and CTTN in 3 colorectal cancer cell lines (HCT-116, SW480 and loVo). GAPDH was used as a loading control. **(B)** Cell migration assays of HCT-116, SW480 and loVo cell lines after co-transfection. The expression data are presented as the mean ± SD of triplicate samples, and asterisks indicate as follows: *P < 0.05. **P < 0.01. **(C)** Cell invasion assays were determined after co-transfection in HCT-116, SW480 and loVo cell lines. Data are presented as mean ± SD and are representative of 3 repeated experiments. *P < 0.05. **P < 0.01.

**Figure 8:**
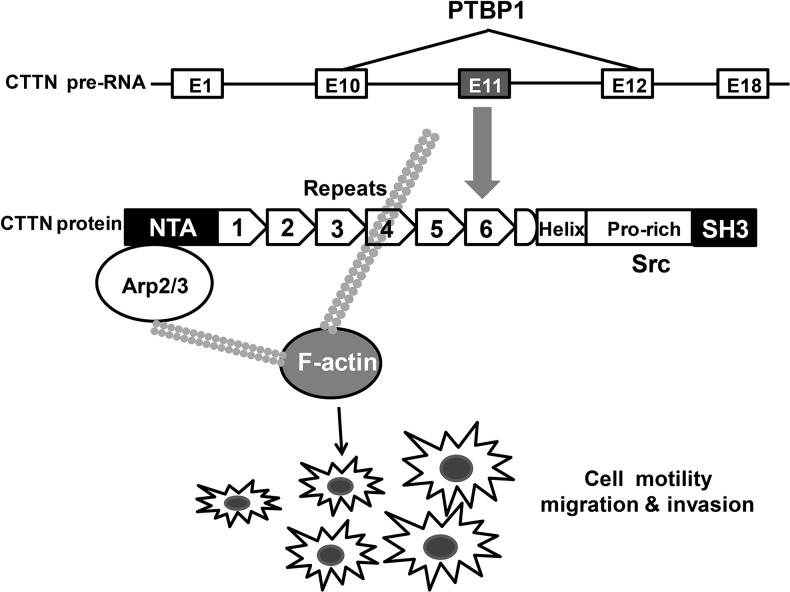
Proposed schema of PTBP1-alternative splicing of CTTN pathway in the study

Influences on cell motility caused by PTBP1 maybe due to the splicing events of exon 11 of *cortactin* gene in colorectal cancer. To further test the above hypothesis, we performed a knockdown of PTBP1 and an over-expression of his/myc-cortactin isoform-a construct or an empty vector control for a rescue study in three colorectal cancer cell lines. Western blot analyses revealed that the PTBP1 siRNA knockdown effect and his/myc-cortactin isoform-a construct increased the cortactin protein (Figure 7A). Real-time PCR and RT-PCR also confirmed the reduction effect of exon 11 in PTBP1 siRNA group after transfection for 48 hours (Figure 6C and 6D). Although MTT did not reveal any significant changes in cell proliferation (data not shown), over-expression of cortactin isoform-a enhanced colorectral cancer cell migration and invasion in the three colon cancer cell lines (Figure 7B and 7C). Thus, as a potential functional RNA-binding protein high PTBP1 resulting in the enclusion of exon 11 of *cortactin* gene promoted cell migration and invasion in CRC (Figure 8).

## DISCUSSION

Our results showed that PTBP1 is highly expressed in colorectal cancer tissues and colonic cancer cell lines comparing with normal colon tissue and normal human colon cell lines, as demonstrated by the immunohistochemistry and western blot analysis (Figure 1), consisting with previous studies [20, 37]. In addition, PTBP1 displayed the more frequent strong immunoreactivity in 77.2% of adenomas with or without atypical hyperplasia, similar with the result in CRC (56.3%). It is suggest the possibility that the PTBP1 may paly an important role in transition of adenomas to colon carcinomas.

Besides PTBP1 is a RNA binding protein and is involved in tumorigenesis, we have also studied the association between PTBP1 expression and clinical outcome. The prognosis of stage II patients with CRC are highly heterogeneous, with five-year relative overall survival (OS) rates ranging from more than 80% (better prognosis) to less than 60% (even worse than the stage III)[1]. Recently, a new concept, high-risk patients with stage II colon cancer, which represents a group of stage II CRC patients with poor prognosis, is introduced in 2012 NCCN (National Comprehensive Cancer Network) guidelines. Adjuvant chemotherapy benefit in stage II colon cancer patients is debated. Therefore, molecular biomarkers are still needed to predict the outcome of stage II CRC patients so that the patient would benefit from an individualized therapy. Here, for the first time we demonstrated that high PTBP1 expression was significantly and independently associated with poor DFS and OS in patients with stages II/III CRC. More importantly, our study also showed that over-expression of PTBP1 was correlated with poor DFS with stage II (without lymph node metastasis) and stage III patients (with lymph node metastasis), respectively and independently (Figure 1B). Thus PTBP1 expression levels had prognostic value for stage II/III CRC patients, especially for stage II. We found the total mRNA levels of EGFR and four EGFR transcripts were dramatically reduced after PTBP1 stable knockdown (Figure 4B and Supplementary Figure 5), which in agreement with previous study that PTBP1 stablized mRNA of EGFR by inhibiting ANXA7-mediated EGFR degradation in glioblastoma [38]. It is indicated that PTBP1 may have impact on targeted therapy of EGFR such as Cetuximab for patients with CRC. Together, we supposed that PTBP1 may be a prognostic marker of stages II/III colon cancers and could be used to guide the invidually targeted therapy.

The functional of PTBP1 significance up-regulation has been studied *in vitro* and *in vivo*. By siRNA mediated knockdown PTBP1, we focused on the knockdown effect in three colon cancer cell lines including one from metastatic site (HCT-116, SW480 and loVo). PTBP1 knockdown inhibited short-term colon cancer cell proliferation in MTT assays, long-term colony formation and by subcutaneous tumor model in nude mice (Figure 2C, 2D and Figure 3) and reduced the invasive capacity of migration and invasion by Wound-healing assays and Transwell Migration/Invasion assays (Figure 2E and 2F), consisting with other results in CRC and tumor cells from multiple origins [19, 20]. PTBP1 siRNA transfection induced late apoptosis by Annexin V/FITC Apoptosis Assay (Supplementary Figure 2), which may contribution to cell proliferation. Together, these results implicated PTBP1 is involved in colorectal cancer progression and may be proposed as a new biomarker of CRC.

The final biological effect of PTBP1 depended on the combination of its different target transcripts, transcription levels of mRNAs, post-transcriptional regulation and the network of genes [10, 11, 39–41]. We detected binding of PTPB1 to the intron 11 of *cortactin* gene in HCT-116 cell by RIP and biotin pull down assays (Figure 5), consistenting with the CLIP-seq results in Hela cells [10]. Alternatively splicing events are particularly relevant in oncology such as cortactin. Cortactin binds to filamentous actin (F-actin) by means of six N-terminal “cortactin repeats” and activates the Arp2/3 complex to regulate cytoskeletal dynamics and cell motility particularly [42, 43]. The cortactin family is composed of at least 3 transcripts: cortactin isoform-a, cortactin isoform-b and cortactin isoform-c. Exon 11 of *cortactin* gene only existed in cortactin isoform-a, which is the most and the most predominant one among all the transcripts [36]. Cortactin isoform-b co-expressed with cortactin isoform-a lacks the 6th repeats (exons 11) is much less abundant, whereas the cortactin isoform-c is fewer [36]. In addition, cells that over-express cortactin isoform-b showed significantly reduced cell migration compared with cortactin isoform-a over-expressing cells [36]. It has been proved that cortactin protein as well as tyrosine phosphorylation of itself which has been reported to enhance cancer cell motility were expressed at high levels in CRC and associated with poor prognosis [35]. Thus, in addition to over-expression and tyrosine phosphorylation, alternative splicing of the F-actin binding domain of cortactin which influences cell migration is one important mechanism contribute to the etiology of cancer.

Identification of *cortactin* mRNA as one of the novel PTPB1 target mRNAs is an interesting finding. PTBP1 and cortactin both have roles as an oncogene in CRC [20, 34, 43]. Our study indicated that knocking down PTBP1 or suppressing *cortactin* could both decrease cell migration and invasion in colon cancer cells (Figure 2 and Supplementary Figure 4) and PTPB1 mediates inclusion of the alternative exon 11 in *cortactin* pre-RNA changing the F-actin binding domain involved in cell motility (Figure 5 and Figure 7D). Recently, a report has formed a pattern of colon-cancer specific alterations including exon 11 of corttactin by exon-microarray [44]. Similarly, our results showed isoform-a (exon 11) of cortactin and the percentage of cortactin isoform-a to all its transcripts was higher in CRC than normal tissues (Figure 6A). In addition, the expression pattern of PTBP1 and cortactin was positive correlated in CRC, as well as PTBP1 and the ratio of cortactin isoform-a to all its transcripts (Figure 6B). Thus the correlation of PTBP1 and cortactin isoform-a up-regulation on cell motility must be further examined. After knocking down of PTBP1, the mRNA level of toltal cortactin didn't change much but cortactin isoform-a decreased (Figure 6C). Our data also supported the notion that over-expression of cortactin isoform-a could rescue the knocking down PTBP1 effect of migration and invasion in 3 colon cancer cell lines (Figure 7) but not affect cell proliferation (data not shown). Together, it is suggested that PTBP1 involved in various biological processes by different target pre-RNAs and it regulated cell motility partly by alternatively splicing of cortactin exon 11.

In conclusion, we identified that PTBP1 over-expression is independently associated with the poor prognosis in patients with stages II/III CRC clinically and knockdown of PTBP1 generally depressed tumorigenesis *in vitro* and *in vivo* by regulating PTBP1 target pre-RNAs. One model is that alternative splicing exon 11 of *cortactin* leading to the changing of F-actin binding domain, by which PTBP1 influences cell migration and invasion.

## MATERIALS AND METHODS

### Patients and tissue samples

The protocol of the present study was approved by the local ethical committee and informed consent was obtained from each patient. Colorectal cancer patients who underwent a radical colectomy at the Peking Union Medical College Hospital, Beijing, between March 2003 and March 2014 were available for the study. Patients who underwent a radical resection for stagesII/III colorectal caner at diagnosis were selected. To investigate specifically the prognostic value of high PTBP1, tissues from patients with stageIcolorectal cancer (very low risk of progression), patients with preoperatively distant metastases (stage IV) and patients who underwent neoadjuvant radiotherapy were excluded. Therefore, in all 158 patients (92 men and 66 women; mean age 62 years, range 21-84 years) with stages II/III colorectal carcinoma tissues, 106 paired adjuvant normal mucosa tissues (≥5cm) were selected. Data on clinical outcome were obtained from patients’ records. The clinicopathological features of the patients examined are summarized in supplementary Table 1. TNM stage was based on the 7th edition of AJCC (American Joint Committee on Cancer) staging system. Overall survival (OS) rate and disease-free survival (DFS) rate were used to evaluate the clinical significance of PTBP1 expression in patients with CRC. The median survival time was 53 months (range: 6 to 94 months). In addition, 44 cases of adenoma with dysplasia lesions or not were also included in this study.

### Immunohistochemical analysis

PTBP1 expression was evaluated by immuno-histochemistry in formalin-fixed, paraffin-embedded tissue sections. The sections were incubated with rabbit polyclonal anti-PTBP1 antibody (1:1000 dilutions, provided by Institute of Basic Medical Sciences, Chinese Academy of Medical Sciences and Peking Union Medical College) at 4°C overnight. Staining was performed using a diaminobenzidine staining kit (ZhongShan Goldenbridge Biotechnology Co., China). For PTBP1 assessment, all sections were scored in a semiquantitative fashion according to the method described previously [45], which considers both the intensity and percentage of cells staining at each intensity. Intensities were classified as 0 (no staining), 1 (weak staining), 2 (distinct staining) and 3 (very strong staining). For each slide, a value designated HSCORE was obtained by application of the following algorithm: HSCORE=∑P*i*(I+1), where I and P*i* represent intensity and percentage cells that stain at each intensity. Our final scores ranged from 0 to 3. Specimens with an HSCORE<2 were regarded as weak/median positive (low-level group), HSCORE ≥2 were regarded as strong expression (high-level group). The scores were evaluated separately by two investigators who are blinded to the clinical parameters.

### Cell lines

Human colorectal carcinoma cell lines HCT-116, SW480, HCT-8, HT-29, DLD1 and loVo (from the Cell Center of Peking Union Medical College) were cultured in Dulbecco's modified Eagle's medium supplemented with 10% fetal bovine serum. Human normal colon epithelial cell line CCD 841 CoN and human normal colon fibroblast cell line CCD-112 CoN (from ATCC) were routinely cultured in recommended media. All cells were maintained at 37°C and 5% CO_2_.

### Western blotting

Tissue protein and cells were lysed with TNTE buffer which contained protease inhibitors and ten micrograms of protein were loaded onto 12% sodium dodecyl sulphate (SDS)-polyacrylamide gels and the detailed procedures have been described previously [46, 47]. The protein samples were analyzed using primary antibodies include polyclonal rabbit anti-PTBP1 (1:3,000; provided by Institute of Basic Medical Sciences, Chinese Academy of Medical Sciences and Peking Union Medical College) and mouse anti-GAPDH (1:3000; Abmart) antibodies.

### The PTBP1 siRNA, cortactin plasmids and transfection

The cDNA target sequences of siRNAs for PTBP1 were purchased from Invitrogen Trading Co.(Supplementary Table 2). Less than 30% cells were seeded per well in six-well culture plates and transiently transfected 2μl for each well at a working concentration of 20 nmol with transfection reagent INTERFERin (PolyPlus-transfection). The knockdown of PTBP1 was measured using western blot after 48-72 hours of transfection. Human cortactin (NM_005231.3) cDNA were cloned into *Kpn*I/*Eco*RI restriction sites of pCDNA4.0/myc-His (−) vector from Beijing Syngentech Co. The plasmids were purified using the EndoFree Plasmid Maxi Kit (QIAGEN) and transfected into colon cancer cell lines using Lipofectamine 2000 (Invitrogen) at a final concentration of 100 to 200 nM.

### MTT assays, colony formation assay, wound-healing assay, migration/invasion assays and annexin V apoptosis assay

A 3-(4,5-Dimethylthiazol-2-yl)-2,5-diphenyltetra-zolium bromide (MTT) cell proliferation assay was use to assess the cell proliferation after transfection, and a colony formation assay were applied and the detaile was as described [48]. For wound-healing assay when the cells reached 90% confluence, a single wound was made in cell monolayer by using a P-200 pipette tip. The percentage of wound healing closure were observed at 0, 24 and 48h under phase-contrast microscope and measured with Image-Pro Plus version 6.0 (Media Cybernetics, Bethesda, USA). Migration and invasion assays were performed in 24-transwell migration chamber (8μm pore) (Corning Life Sciences, Acton, MA), coated with BD MatrigelTM Basement Membrane Matrix (BD Biosciences, San Jose, CA) (1:1dilution, 50μl/cm^2^) or not. After 20 to 48 hours, transwell chambers were fixed and stained with crystal violet. The cells on lower surface of the filters were counted in three microscopic fields per well. According to the manufacturer's instructions, annexin V:FITC and propidium iodide (PI) were added to the suspension (Annexin V-FITC kit, BD Biosciences). The dual-fluorescence was analyzed using Accuri C6 flow cytometer.

### Murine tumor model

HCT-116 cells were infected with adenovirus (AD)-control shRNA and AD-PTBP1 shRNA. The 2 target sequences of PTBP1 were AD-PTBP1 shRNA (5′-ACCGCAAGATGGCACTGATCCAGAT-3′) and AD-PTBP1 shRNA* (5′-UGACAAGAGCCGUGACUAC-3′). Knockdown of PTBP1 was confirmed by western blot. The multiplicity of infection (MOI) was 50. HCT-116 cells (10^7^) in 100 μl of a 1:1 mixture of culture medium were implanted subcutaneously into the four-week-old BALB/C-nude mices into AD-control shRNA and AD-PTBP1 shRNA treatment groups (n = 10 tumors per group). Tumors were measured every 4 days and tumor volume (V) was estimated using a model V = (L × W2)/2 (L: length;W: width).

### Lentiviral transfection

Lentiviral clones expressing PTBP1 shRNA or non-targeting control shRNA were acquired from Thermo Fisher. The target sequences of PTBP1 were lenti-PTBP1 shRNA (ATTGTTGTACTTGACGTTGAG). Viral particles were produced in 293T cells with the PLKO.1 set of helper plasmids (System Biosciences) in DMEM (Dulbecco's modification of Eagle's medium Dulbecco) media. Viral stocks were concentrated by precipitation with PEG-8,000 and titered according to the manufacturer's instructions. Lentiviral transduction to engineer pure populations of Puro resistant cells stably expressing PTBP1-directed or control shRNAs (shPLKO.1). One of two shPTBP1 clones displayed high efficiency of knockdown (nearly 80% reduction of mRNA) and were used for all related experiments.

### RNA extraction and Real-time PCR and RT-PCR analyses

Total RNA was extracted from tissues using TRIzol Reagent and reverses transcription and was reverse transcribed using a reverse transcription system to generate a cDNA template according to the manufacturer's instructions. Real-time PCR were performed as described previously with GAPDH as an internal control for normalization [49]. PCR was performed after reverse transcription. Primer sequences are listed in Supplementary Table 4.

### RNA IP

RNA IP was performed in HCT-116 cells using the RiboCluster Profiler RIP-Assay Kit (MBL International). RIP-certified polyclonal PTBP1 antibody (MBL International) and Protein A Sepharose CL-4B beads (GE Healthcare) was used.

### Biotin pull-down

RNA probes of fragments located in the intron 10 or intron 11 of cortactin gene were prepared from pGEM-3zf vector. The primers are shown in Supplemental Table 5. Cell extracts were isolated from HCT-116 cells using nuclear protein extraction reagents (Thermo Scientific). The reaction mixture contained 150 mg of the cell extracts and 2μg of each biotinylated RNA probe. RNA affinity capture was subsequently conducted with streptavidin-sepharose beads as described previously [50].

### Statistics analysis

Analyses were performed using SPSS version 19.0 (SPSS, Inc., Chicago, IL, USA). Group comparisons for continuous data were compared by Student t-test. Pearson's χ^2^ test or Continuity Correction test were used to analyze the association between nuclear PTBP1 status and patient characteristics with PTBP1 HSCORE. Kaplan–Meier method and Log-rank analysis were used for survival analysis and Cox hazard proportional model were used to adjust for covariate effects on the OR (odds ratio). *In vitro* and *in vivo* experiments, results were presented in the text as mean ± standard deviation (SD). For each test, *P* < 0.05 was considered statistically significant.

## SUPPLEMENTARY MATERIALS FIGURES AND TABLES






