# Supplementary material for: High expression of PTBP1 promote invasion of colorectal cancer by alternative splicing of cortactin
Source: Oncotarget. 2017 Mar 3;8(22):36185–202. doi: 10.18632/oncotarget.15873 (PMC5482648; doi:10.18632/oncotarget.15873)
Supplement: Supplementary file 1 [file oncotarget-08-36185-s001.pdf]

High expression of PTBP1 promote invasion of colorectal cancer by alternative splicing of cortactin

SUPPLEMENTARY FIGURES AND TABLES

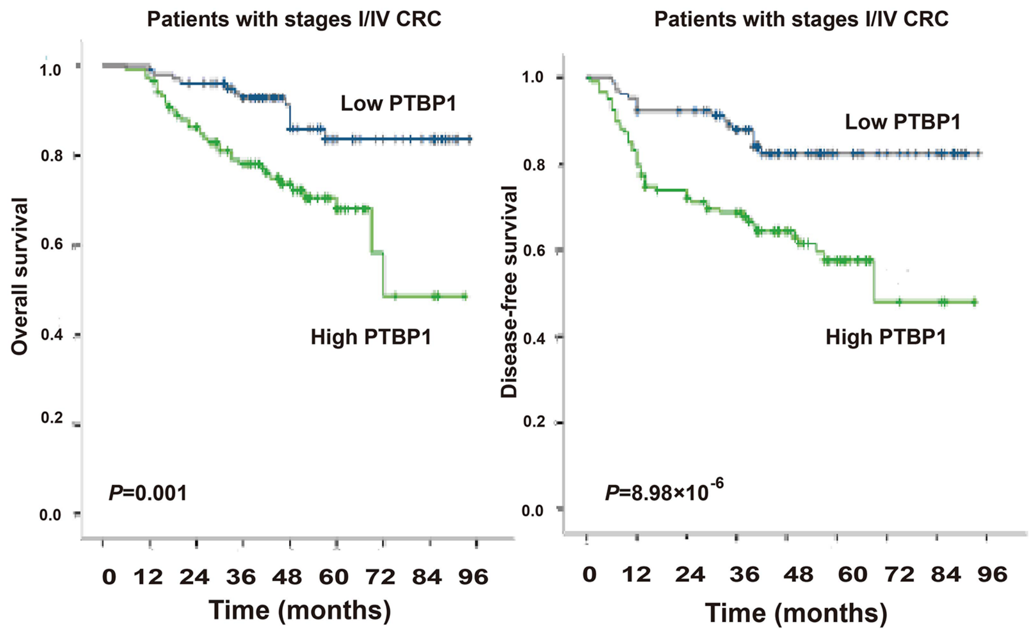

Supplementary Figure 1: Overall survival and disease-free survival curves for patients according to the expression levels of immunohistochemical variables in 202 stage I-IV CRC patients.

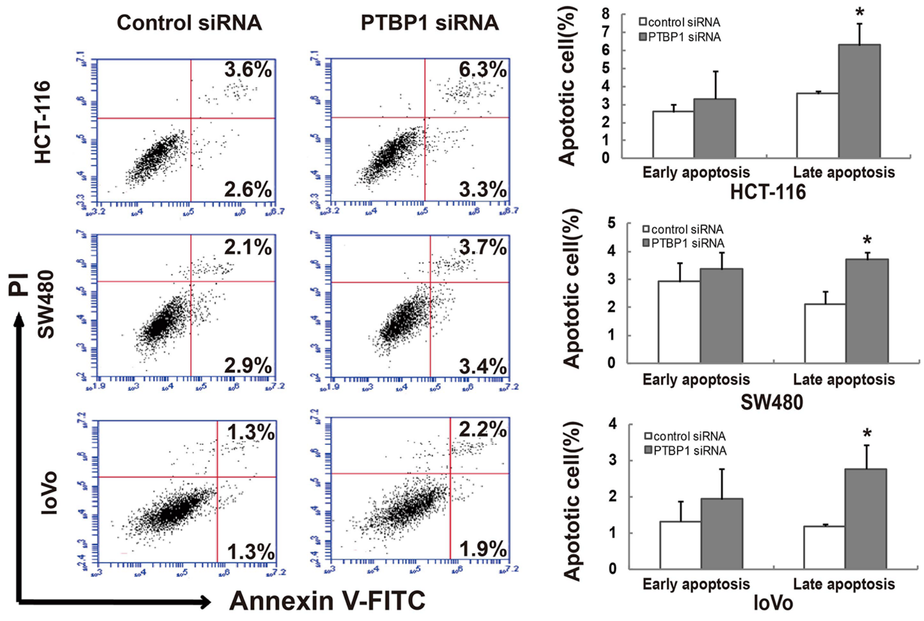

Supplementary Figure 2: Kockdown of PTBP1 affects tumor cell late apoptosis Apoptosis was evaluated at 72 h after tansfection staining with Annexin-V and PI. Flow cytometry profile represents Annexin-V-FITC staining in x axis and PI in y axis. The number represents the percentage of early apoptotic cells (lower right quadrant) and late apoptotic cells (higher right quadrant). The experiment was repeated three times and data represent the average of the early apoptotic and late apoptotic cells. Expression data are presented as the mean ± SD of triplicate samples. *$P<0.05$.

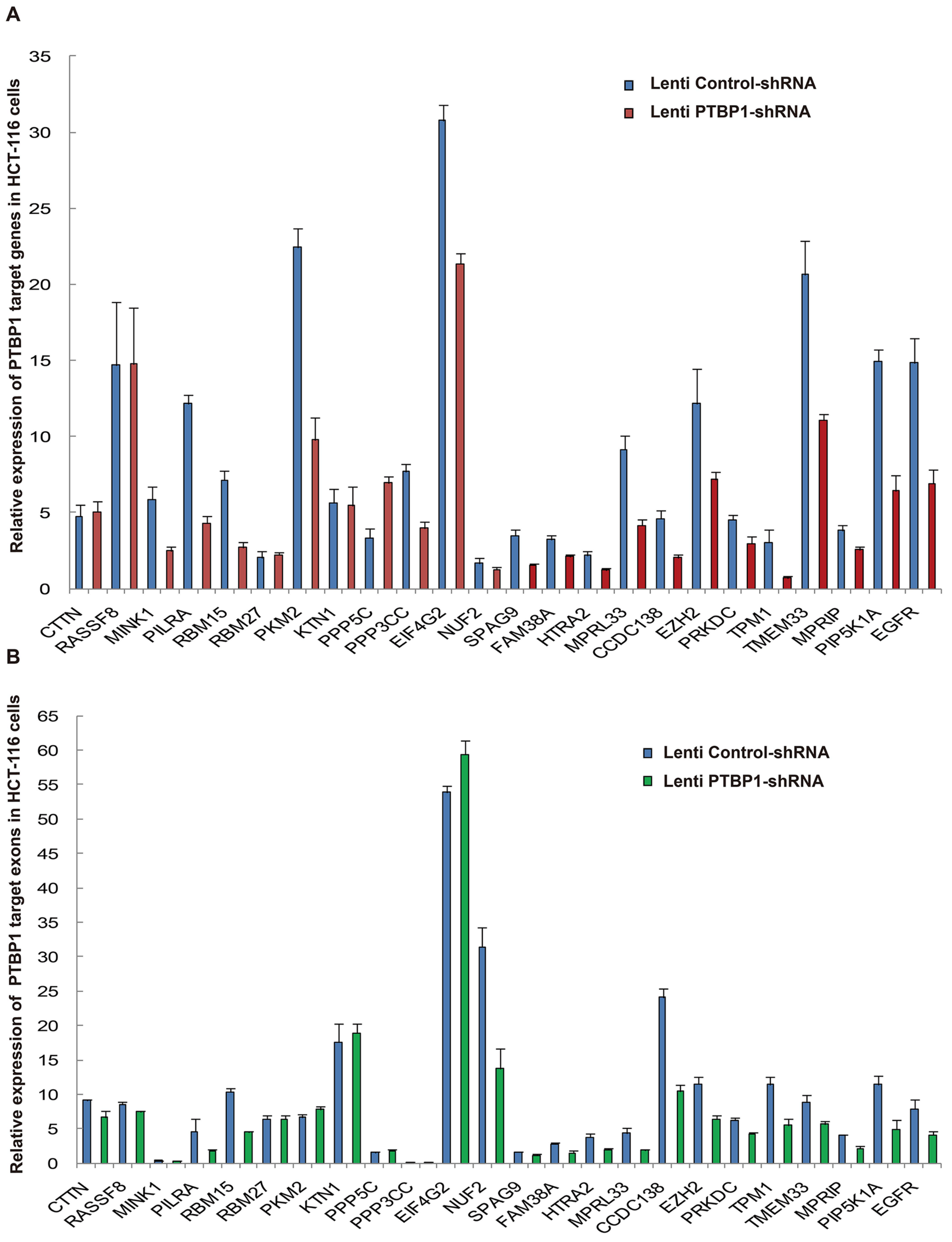

Supplementary Figure 3: The levels of mRNAs **(A)** and each exon **(B)** changes of PTBP1 target genes by quantitative real time PCR in HCT-116 cells after lentiviral-mediated PTBP1-shRNA knockdown. Data are mean ± SD of triplicate samples.

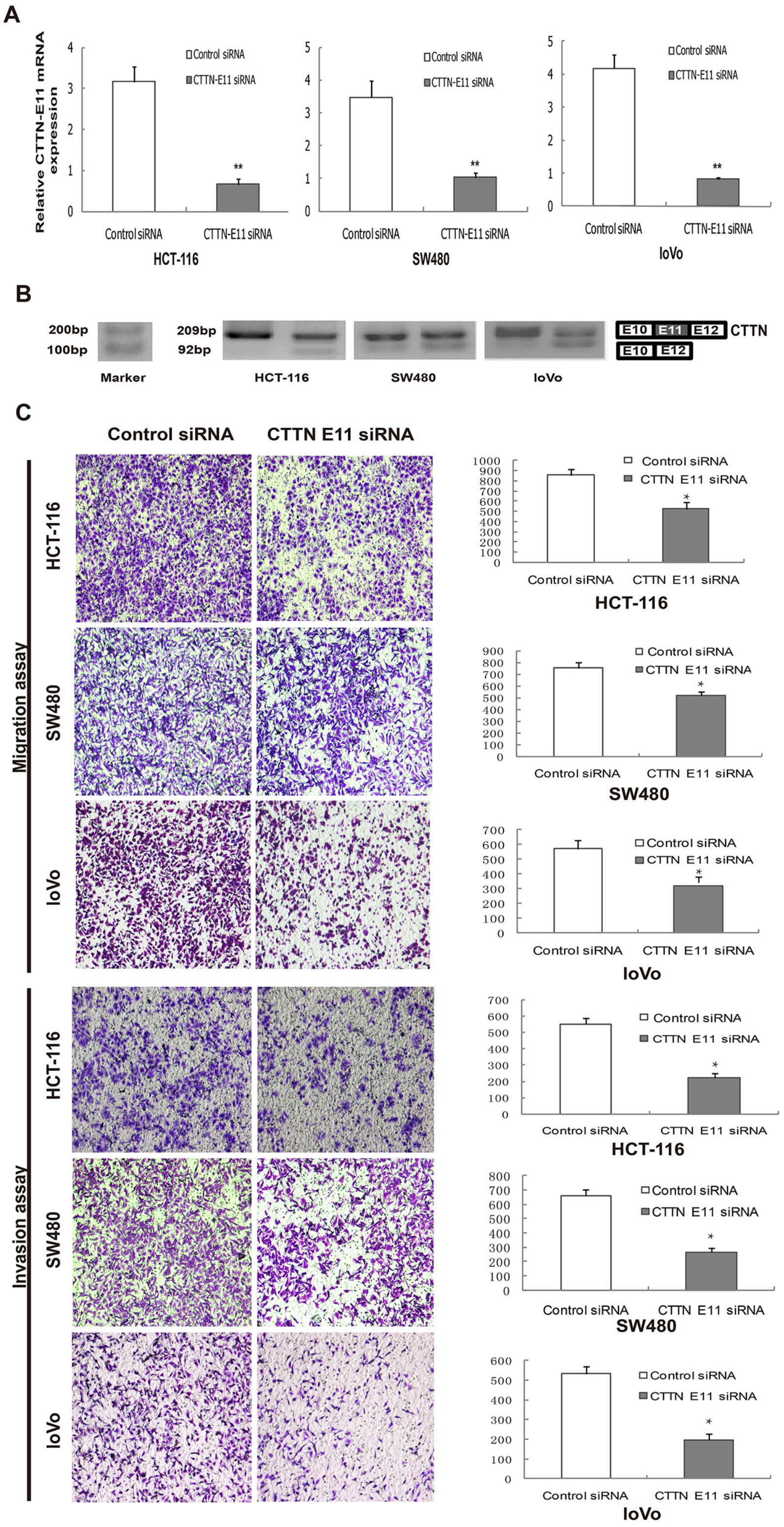

Supplementary Figure 4: Real-time PCR **(A)** and RT-PCR **(B)** demonstrating significant siRNA-mediated knockdown of CTTN exon 11 after 48 hours post-transfection in three colorectal caner cell lines (HCT-116, SW480 and loVo). GAPDH was used as control. Data are presented as mean ± SD of triplicate times. $^{**}P < 0.01$. **(C)** Cell migration and invasion assays were determined 24-48 hours after transfection. Photos were taken under inverse microscope (Nikon, Eclipse, TE2000-U), 10×. Data are presented as mean±SD and are representative of 3 repeated experiments. $^{**}P < 0.01$.

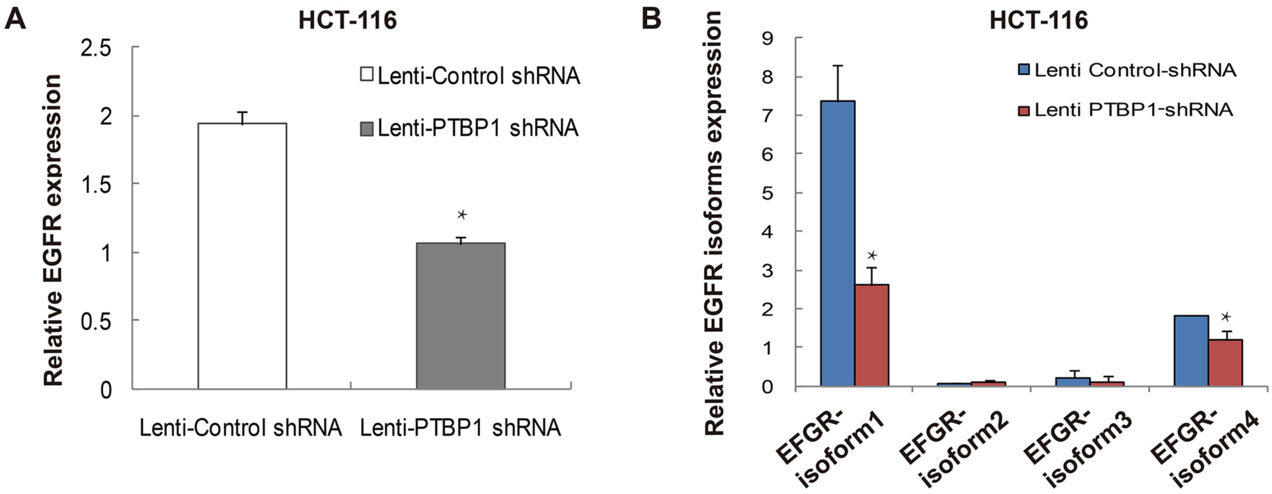

Supplementary Figure 5: Relative mRNA expression of EGFR and four EGFR transcripts in HCT-116 cells after lentiviral-mediated PTBP1-shRNA knockdown. Data are mean ± SD of 3 separate times. *$P < 0.05$.

Supplementary Table 1: Clinicopathological features of the 202 Patients with Colorectal Cancer

Clinicopathological features	No. of patients	%
Age,years		
<60	78	38.6
≥60	124	61.4
Gender		
Male	119	58.9
Female	83	41.1
Preoperative CEA*,ng/ml		
CEA≤5	69	41.8
CEA>5	96	58.2
Tumor site		
Colon	99	49
Rectum	103	51
Tumor size, cm		
<5	106	52.5
≥5	96	47.5
Histological type		
Adenocarcinoma	168	83.2
Others*	34	16.8
Tumor grade*		
G1/G2	156	77.2
G3	46	22.8
Tumor status		
T1 and T2	23	11.4
T3 and T4	179	88.6
Nodal status*		
N0	101	50
N1/N2	101	50
TNM stage		
I	17	8.4
II	75	37.1
III	83	41.1
IV	27	13.4
Disease status		
Alive with disease	16	7.9
Alive without disease	123	60.9
Dead from disease	59	29.2
Dead from other disease	4	2

Note: *The number of patients who have been tested preoperative CEA was 165, the toltal number of other featues was 202. Others=mucinous or signet-ring cell carcinoma. Tumor grade: G1=well differentiated, G2=moderately differentiated, G3=poorly differentiated. Nodal status: N0=no lymphnode involvement. N1=1-3 nodes involved. N2=more than or equal to four nodes involved.

Supplementary Table 2: The Sequences of siRNAs

**Control siRNA**	Sense	5’- AACCUAGAAGGACCGUUUGUG-3’
	Anti-sense	5’- CACAAACGGUCCUUCUAGGUU-3’
**PTBP1 siRNA**	Sense	5’-AGAAGGACCGCAAGAUGGCACUGAU-3’
	Anti-sense	5’-AUCAGUGCCAUCUUGCGGUCCUUCU-3’
**PTBP1 siRNA***	Sense	5’-ACCGCAAGAUGGCACUGAUCCAGAU-3’
	Anti-sense	5’-AUCUGGAUCAGUGCCAUCUUGCGGU-3’

Note: *Another siRNA targeting differente site to avoid off target effects.

Supplementary Table 3: Correlation of Clinicopathologic Features with PTBP1 in 202 Patients with Colorectal Cancer.

See Supplementary File 1

Supplementary Table 4: All the Primers in the study.

See Supplementary File 2
