# Supplementary material for: High expression of PTBP1 promote invasion of colorectal cancer by alternative splicing of cortactin
Source: Oncotarget. 2017 Mar 3;8(22):36185–202. doi: 10.18632/oncotarget.15873 (PMC5482648; doi:10.18632/oncotarget.15873)
Supplement: Supplementary file 2 [file oncotarget-08-36185-s002.docx]

**Supplementary Table 3. Correlation of Clinicopathologic Features with PTBP1 in 202 Patients with Colorectal Cancer**

| Clinicopathologic Features | Case | PTBP1 expression | | | | *P** |
| --- | --- | --- | --- | --- | --- | --- |
|  |  | Weak/Median levels(%) | | High levels(%) | |  |
| Case | 202 | 89 | 113 | |  | |
| Age, years |  |  |  | |  | |
| ＜60 | 78 | 37(18.3%) | 41(20.3%) | | 0.44 | |
| ≥60 | 124 | 52(25.7%) | 72(35.6%) | |  |  |
| Gender |  |  |  | |  | |
| Male | 119 | 54(26.7%) | 65(32.2%) | | 0.65 | |
| Female | 83 | 35(17.3%) | 48(23.8%) | |  |  |
| Preoperative CEA*,ng/ml |  |  |  | |  | |
| CEA≤5 | 96 | 42(25.5%) | 54(32.7%) | | 0.68 | |
| CEA＞5 | 69 | 28(17.0%) | 41(24.8%) | |  |  |
| Tumor site |  |  |  | |  | |
| Colon | 99 | 38(18.8%) | 61(30.2%) | | 0.11 | |
| Rectum | 103 | 51(25.2%) | 52(2.7%) | |  |  |
| Tumor size, cm |  |  |  | |  | |
| ＜5 | 106 | 20(9.9%) | 86(42.6%) | | 0.38 | |
| ≥5 | 96 | 23(11.4%) | 73(36.1%) | |  |  |
| Histological type |  |  |  | |  | |
| Adenocarcinoma | 168 | 74(36.6%) | 94(46.5%) | | 0.99 | |
| Others | 34 | 15(7.4%) | 19(9.4%) | |  |  |
| Grade |  |  |  | |  | |
| G1/G2 | 156 | 68(33.7%) | 88(43.6%) | | 0.8 | |
| G3 | 46 | 21(10.4%) | 25(12.4%) | |  |  |
| Tumor status |  |  |  | |  | |
| T1 and T2 | 23 | 11(5.4%) | 12(5.9%) | | 0.7 | |
| T3 and T4 | 179 | 78(38.6%) | 101(50.0%) | |  |  |
| Nodal status |  |  |  | |  | |
| N0 | 101 | 23(11.4%) | 78(38.8%) | | 0.63 | |
| N1/N2 | 100 | 20(10.0%) | 80(39.8%) | |  |  |
| Metastasis |  |  |  | |  | |
| M0 | 176 | 77(38.1%) | 99(49.0%) | | 0.82 | |
| M1 | 26 | 12(5.9%) | 14(6.9%) | |  |  |
| TNM Stage |  |  |  | |  | |
| Ⅰ | 17 | 7(3.5%) | 10(5.0%) | | 0.55 | |
| Ⅱ | 75 | 37(18.3%) | 38(18.8%) | |  |  |
| Ⅲ | 83 | 32(15.8%) | 51(25.2%) | |  |  |
| Ⅳ | 27 | 13(6.4%) | 14(6.9%) | |  |  |

Note: *The number of patients who have been tested preoperative CEA was 165, the toltal number of other featues was 202. *P* value, Pearson’s χ^2^ test.
