# Supplementary material for: High expression of PTBP1 promote invasion of colorectal cancer by alternative splicing of cortactin
Source: Oncotarget. 2017 Mar 3;8(22):36185–202. doi: 10.18632/oncotarget.15873 (PMC5482648; doi:10.18632/oncotarget.15873)
Supplement: Supplementary file 3 [file oncotarget-08-36185-s003.doc]

**Supplementary Table 4. All the Primers in the study.**

| **Primers** | **Primer Sequences 5’-3’** |  | |
| --- | --- | --- | --- |
| **Primers for Real-time PCR of PTBP1 detection** | |  | |
| q-PTBP1-F | ATTCTTTTCGGCGTCTACGGT | |  |
| q-PTBP1-R | TATTGAACAGGATCTTCACGCG | |  |
| q-GAPDH-F | CATCCATGACAACTTTGGTATC | |  |
| q-GAPDH-R | CCATCACGCCACAGTTTC | |  |
| **Primers for RT-PCR of PTBP1 target genes** | |  | |
| KE-CTTN-E10-F | GACAGACAGACAAGACAA |  | |
| KE-CTTN-E10-R | CGAATCCTTTGGAGTAGT |  | |
| KE-MRPL33-E3-F | TTTTGGCCGGTGACGGAG |  | |
| KE-MRPL33-E3-R | GCCCTCAGTCTTCTCTTTA |  | |
| KE-HTRA2-E7-F | GGAGGTGATTGGAGTGAA |  | |
| KE-HTRA2-E7-R | GTCACATATAAGGTCAGT |  | |
| KE-CCDC138-E14-F | TGGACTTGAAGACAGAAGAA |  | |
| KE-CCDC138-E14-R | GAAGCATCAGATGAATCGTAA |  | |
| KE-EZH2-E19-F | AGAATGGAAACAGCGAAGGAT |  | |
| KE-EZH2-E19-R | AGGGCATTCACCAACTCCACAAA |  | |
| KE-PPP3CC-E15-F | CACTCCCTCTGGGCGTCCTCT |  | |
| KE-PPP3CC-E15-R | TCTTCCCTTGGTCGCTCCTGT |  | |
| KE-CTTN-E5-F | AATGATGTGAGTGAGAAG |  | |
| KE-CTTN-E5-R | CTTATCCATTCGGTCTTG |  | |
| **Primers for real time-PCR of PTBP1 target genes** | |  | |
| q-CTTN-F | AAGGATCGGATGGATAAG |  | |
| q-CTTN-R | GTACTGTCTTCTGGTAGG |  | |
| q-EZH2-F | GGATACAGACAGTGATAG |  | |
| q-EZH2-R | TTCTTCTCTTCTTCTTCTT |  | |
| q-PILRA-F | TGAGAATATCCTGGAGAC |  | |
| q-PILRA-R | ACATAATCCTTGTGAATGG |  | |
| q-RASSF8-F | GATGGAGTTCAGAGGATT |  | |
| q-RASSF8-R | CTATTGCTTGAGCTAAGG |  | |
| q-MPRIP-F | CAGATGGGACCGACTTTG |  | |
| q-MPRIP-R | TGCTCGTAAAGGATGAAGAA |  | |
| q-SPAG9-F | ACTATCACCTCTTAGACCTT |  | |
| q-SPAG9-R | TATAGCCACACCAGACTT |  | |
| q-NUF2-F | TGTGATTCATATTCGCAAT |  | |
| q-NUF2-R | ACTATTTGTAAGGCTCTCA |  | |
| q-KTN1-F | GAACAAGACTTCCAAGGA |  | |
| q-KTN1-R | AACCATTCACCATAACTCA |  | |
| q-PPP5C-F | AAGGTGAAGCCCCATGACAAG |  | |
| q-PPP5C-R | CGATGGCCCGCTCAAAGG |  | |
| q-TMEM33-F | ACCTCTTCCTTCCTTAATCTC |  | |
| q-TMEM33-R | CGTGGCTACTTCTTCCTT |  | |
| q-TPM1-F | CAGCAGATGAGAGTGAGA |  | |
| q-TPM1-R | GCCTCTTTCAGTTGGATC |  | |
| q-PKM2-F | CACATTCCTGGAGCACAT |  | |
| q-PKM2-R | CAGATGATGCCAGTGTTC |  | |
| q-PPP3CC-F | GACTGGAAGAGGAAGTAG |  | |
| q-PPP3CC-R | CTGTGATTGGAGCATCTA |  | |
| q-CCDC138-F | CCTAGATGATGAACTGGATT |  | |
| q-CCDC138-R | CGAGGTACTAACTCTATAATCA |  | |
| q-PRKDC-F | CTATGCTGGCTGGACCTG |  | |
| q-PRKDC-R | CGCTCCTACAGTTCTCTC |  | |
| q-RBM15-F | GAATACAAGACTCTGAAGATAA |  | |
| q-RBM15-R | ACACTTACATCACCGAAG |  | |
| q-RBM27-F | GTTGTAGCACTGGTCAAG |  | |
| q-RBM27-R | GTGGAAGGTAGTTCTTAGTATAG |  | |
| q-FAM38A-F | GCTGGAGGAGGAGTTGTA |  | |
| q-FAM38A-R | TCACGAGTCCACTTGATC |  | |
| q-MRPL33-F | AATCCTGTAGCGTGTAAT |  | |
| q-MRPL33-R | CTGTCCTAATATGAAGAACTT |  | |
| q-MINK1-F | GGACGAGGAGGAAGAGAT |  | |
| q-MINK1-R | TAGTAGGTGGCGATGTTG |  | |
| q-EIF4G2-F | ATTCATCCTCTGCTCCTT |  | |
| q-EIF4G2-R | GGCACTGACTTGTAGATC |  | |
| q-HTRA2-F | GGAGTCAGTACAACTTCATC |  | |
| q-HTRA2-R | GGTCCAGGATCTCGATATAG |  | |
| q-PIP5K1A-F | ACTCCTGTAATCTCTGTAA |  | |
| q-PIP5K1A-R | TCTACGAATAGGCATCTT |  | |
| **Primers for real time-PCR of PTBP1 target genes (Exon of alternative splicing according to previous study^*^)** | |  | |
| q-CTTN-Exon11-F | TATAAGACTGGTTTTGGA |  | |
| q-CTTN-Exon11-R | TTCTCCTTGTAATCAAAC |  | |
| q-RASSF8-Exon2-F | GCCTGTGTAGATCATCCTAGAATA |  | |
| q-RASSF8-Exon2-R | TCCGAGCCAGTGAGAATG |  | |
| q-MINK1-Exon17-F | CCTGGGAATAAAGCCAAG |  | |
| q-MINK1-Exon17-R | ACCAATTGCTCGCTTATAG |  | |
| q-PILRA-Exon3-F | AGACTCTTGGCACATAAG |  | |
| q-PILRA-Exon3-R | AGATCAGTCCCAAAATCAT |  | |
| q-RBM15-Exon2-F | CAGATAGGAGTTAGGTATGAGA |  | |
| q-RBM15-Exon2-R | GGACACCACTATAACCACTA |  | |
| q-RBM27-Exon9-F | TCTGTTGGCAGCTCGTTT |  | |
| q-RBM27-Exon9-R | CTGGAGACTGAGGTATGGTATC |  | |
| q-PKM2-Exon3-F | CTCAAGTCACTCCACAGA |  | |
| q-PKM2-Exon3-R | ACTCCGTCAGAACTATCAA |  | |
| q-KTN1-Exon2-F | TGGTGTGACCTTGGCTTG |  | |
| q-KTN1-Exon2-R | TACTTAGGAGGCTGAGGCAAT |  | |
| q-PPP5C-Exon5-F | AATGTGCCTACCAGATTC |  | |
| q-PPP5C-Exon5-R | TCTTTGAGTGTGGTTTCC |  | |
| q-PPP3CC-Exon13-F | ACAGTAGAAGCGGTAGAG |  | |
| q-PPP3CC-Exon13-R | TCATTAATTCGGTCCAGAC |  | |
| q-EIF4G2-Exon9-F | CTACCATAACCAGAGTCA |  | |
| q-EIF4G2-Exon9-R | AAGCTGTCCTTTCTTAGA |  | |
| q-NUF2-Exon11-F | GTGGTGGAGAAATATGAA |  | |
| q-NUF2-Exon11-R | CCTTTAAGATACTGGCTAA |  | |
| q-SPAG9-Exon4-F | GCTCTTATGGTGTCTTGTAA |  | |
| q-SPAG9-Exon4-R | TACGGTTTCTGTCAATGG |  | |
| q-FAM38A-Exon8-F | CATGAAGATTTGCCCGTTT |  | |
| q-FAM38A-Exon8-R | CTCAAAGTGGATCAAAGAGC |  | |
| q-HTRA2-Exon7-F | TGAACTACAGCTTCGAGAAC |  | |
| q-HTRA2-Exon-7R | GAGCCCAGGATGACTTTATG |  | |
| q-MRPL33-Exon3-F | TGAGAATGGTGAGCGAAG |  | |
| q-MRPL33-Exon3-R | TCATAATGCAAAAGAGTCAGTTT |  | |
| q-CCDC138-Exon14-F | GCCTGTAGCAACTCTTTA |  | |
| q-CCDC138-Exon14-R | TGATTTTGGAAAGTTTCTGT |  | |
| q-EZH2-Exon17-F | TTTCCAACACAAGTCATCC |  | |
| q-EZH2-Exon-17R | GGGAGACCAAGAATACATTATG |  | |
| q-PRKDC-Exon7-F | GGATTAATTGAGTGGCTTGA |  | |
| q-PRKDC-Exon7-R | CTTCTCCTCTTGGGACAT |  | |
| q-TPM1-Exon6-F | CAAATGTGCCGAGCTTGAAGA |  | |
| q-TPM1-Exon6-R | TTCTCAGCCTGAGCCTCC |  | |
| q-TMEM33-Exon3-F | ACTTCCAGTTAAGCAGAG |  | |
| q-TMEM33-Exon3-R | ATGAGTGAATACAACAGGTA |  | |
| q-MPRIP-Exon9-F | CGAAGAGCCAAGTCACTG |  | |
| q-MPRIP-Exon9-R | TTAGTCAGCCAGCCTTTC |  | |
| q-PIP5K1A-Exon12-F | AACTCCTGCATTACTTACCA |  | |
| q-PIP5K1A-Exon12-R | CTCCACTTCTGCCTTTGT |  | |
| q-EGFR-Exon4-F | CCTGTGCAACGTGGAGAG |  | |
| q-EGFR-Exon4-R | AGGTGGTTCTGGAAGTCC |  | |
| **Primers for EGFR and EGFR transcripts** | |  | |
| q-EGFR-F | CCCATGAGAAATTTACAG |  | |
| q-EGFR-R | GATGATTTTGGTCAGTTT |  | |
| q-EGFRv1-F | CAAACTGCACCTACGGAT |  | |
| q-EGFRv1-R | CGATGGACGGGATCTTAG |  | |
| q-EGFRv2-F | CCTACGGGTCCTAATAAATC |  | |
| q-EGFRv2-R | TTGAAGCAAAGGGAGAAAT |  | |
| q-EGFRv3-F | CGTAAAGGAAATCACAGGTT |  | |
| q-EGFRv3-R | CCAAGGGAACAGGAAATATG |  | |
| q-EGFRv4-F | ACTGGCTGCTTTGTTCAA |  | |
| q-EGFRv4-R | GGATGTTGGATTGGTGGTTA |  | |
| **Primers for biotin pull-down** | |  | |
| CTTNin10-1F | AAGCTTTGCTTCAGCCTCCCAAGTA |  | |
| CTTNin10-1R | GGTACCTGAGAAAGACTCGGTTCCAA |  | |
| CTTNin10-2F | AAGCTTCACTATGTGTCCAGCAACAAC |  | |
| CTTNin10-2R | GGTACCTCTCACCTCCACTCCTACCTT |  | |
| CTTNin11-1F | AAGCTTGGAGGTGAGAATGGACTTACTT |  | |
| CTTNin11-1R | GGTACCTCAGCAGCATCCTTGGTAT |  | |
| CTTNin11-2F | AAGCTTCGTCACTTGTTTATGTTGGA |  | |
| CTTNin11-2R | GGTACCTCTGGTACAGCATGGAATCT |  | |
| nonsense-F | AAGCTTTGGCTCTTAAGCTATTTCTTCCCTTG |  | |
| nonsense-R | GGATCCGTTTCTGGGCTAATATCCACTTATCAG |  | |
| positive-F | CCGGAATTCCCCCCCAGTGAAACTTAGAAGCAGCAAACCACGATCA |  | |
| positive-R | CCCAAGCTTGTTTAGCTGTGTTAAGGGTCAAG |  | |
| **Primers for CTTN and CTTN transcripts** | |  | |
| q-CTTN all-F | AAGGAGAAGGAACTTGAA |  | |
| q-CTTN all-R | ACTTATCCATTCGGTCTT |  | |
| q-CTTNisoform-a(E11)-F | AAGTTAACCCGGAGCTAAGT |  | |
| q-CTTNisoform-a(E11)-R | ACGGTATGACATAGGCAGAT |  | |

*Note: Xue Y, Zhou Y, Wu T, et al. Genome-wide analysis of PTB-RNA interactions reveals a strategy used by the general splicing repressor to modulate exon inclusion or skipping. Mol Cell 2009;36:996-1006. Known Gene exon in UCSC genome database (hg18).
